# Knowledge, attitudes, and behaviors toward the planetary health diet in France: a survey among patients, healthcare professionals, and farmers-bakers’ customers

**DOI:** 10.1186/s12937-026-01300-2

**Published:** 2026-02-27

**Authors:** Florence Carrouel, Rita Nugem, Yohan Fayet, Laurie Fraticelli, Lama Basbous, Jean-Francois Vian, Camille Vindras, Corélie Salque, Claude Dussart, Marie-Thérèse Charreyre, Romain Lan, Audrey Murat-Ringot

**Affiliations:** 1 Laboratory “Health, Systemic, Process” (P2S), UR4129, University Claude Bernard Lyon 1, Lyon, 69008 France; 2 Department of Geography, Université Clermont Auvergne, AgroParisTech, INRAE, VetAgroSup, Territoires, Clermont-Ferrand, 63000 France; 3 Hospices Civils de Lyon, Lyon, 69002 France; 4 Agroecology and Environment Research Unit, ISARA, Lyon, France; 5 Amaranthus,independent researcher, 977 Tizin, Tullins, 38210 France; 6 Ingénierie des Matériaux Polymères, Universite Claude Bernard Lyon 1, INSA Lyon, Université Jean Monnet, Centre National de la Recherche Scientifique (CNRS), UMR 5223, Villeurbanne Cédex, 69622 France; 7 Laboratory Anthropologie Bio-Culturelle, Ethique et Santé (ADES), Aix Marseille University, Centre National de la Recherche Scientifique (CNRS), Etablissement Français du Sang (EFS), Droit, Marseille, 13005 France

**Keywords:** Planetary Health Diet, sustainable nutrition, knowledge-attitude-behavior, food systems, France, dietary transition, cross-sectional survey

## Abstract

**Background:**

The Planetary Health Diet (PHD), developed by the EAT–Lancet Commission, provides a comprehensive framework to promote human and planetary health through dietary transformation. Despite its benefits, adoption of the PHD remains limited in high-income countries like France. This study aimed to evaluate knowledge, attitudes, and behaviors (KAB) related to the PHD across diverse French populations and identify determinants of inadequate KAB.

**Methods:**

This cross-sectional quantitative study was conducted between April and June 2024 under the BIOQUALIM protocol. Eligible participants were adults (≥ 18 years) fluent in French and included healthcare professionals and patients from the Hospices Civils de Lyon (HCL), as well as farmers-bakers’ customers from the Rhône-Alpes region. A validated 89-item questionnaire was administered via paper and digital formats using a convenience sampling strategy. KAB levels were calculated from a structured scoring system. Descriptive statistics and multivariate logistic regressions were used to assess KAB levels and their sociodemographic determinants.

**Results:**

Among 10,500 individuals approached, 1,388 completed the survey (79.6% HCL professionals, 6.4% patients, 14% farmers-bakers’ customers). Only 3% demonstrated an adequate overall KAB score; knowledge adequacy was observed in 61%, while attitude and behavior adequacy remained critically low (3.5% and 0.2%, respectively). Inadequate KAB was significantly associated with omnivorous diets (OR = 3.38), female gender (OR = 2.54), patient status (OR = 13.84), while low food budgets increased risk (≤ 50 €/week, reference OR = 1) and higher weekly expenses were protective (OR range 0.42–0.57). Protective factors included older age (≥ 60 years; OR = 0.17) and higher weekly food expenses (>€100).

**Conclusions:**

Although awareness of sustainable diets is rising, behavioral adherence to the PHD remains poor, highlighting a pronounced knowledge–action gap. Cultural norms, economic limitations, and institutional structures still delay dietary change. Policy efforts must extend beyond education to include economic answers, culturally adapted messages, and systemic reforms to enable a fair and sustainable food transition.

**Supplementary Information:**

The online version contains supplementary material available at 10.1186/s12937-026-01300-2.

## Background

Non-communicable diseases (NCDs), such as cardiovascular diseases, diabetes, and cancer, account for 74% of global deaths, representing a significant public health burden [1]. Cancer has become the leading cause of mortality worldwide, with 9.7 million deaths and nearly 20 million new cases recorded in 2022. Over a lifetime, one in five people will develop cancer [1]. Modifiable risk factors mediating systemic determinants, among which dietary habits, play a critical role in preventing NCDs and several cancer types, including colorectal, breast, prostate, and lung cancers [1]. According to the World Health Organization (WHO), targeted interventions promoting healthy eating behaviors are essential [2]. A balanced diet rich in fruits, vegetables, whole grains, legumes, and plant-based proteins, while limiting added sugars, saturated fats, salt, and additives, characteristics of ultra-processed foods, has proven effective in preventing NCDs, as demonstrated by dietary patterns like the Mediterranean and DASH diets [3]. 

In this context, the Planetary Health Diet (PHD), proposed by the EAT-Lancet Commission, emerges as a holistic solution integrating the One Health concept to promote human, animal, and environmental health [4]. This dietary model prioritizes plant-based foods and limits red meat and ultra-processed products, thereby reducing the risk of NCDs. Considering animal health, PHD reduces demand for intensive livestock farming, reducing animal suffering and minimizing the environmental impact of the meat industry. Furthermore, the PHD aims for low environmental impact by reducing greenhouse gas emissions, water consumption, and land use, while supporting sustainable agriculture and preserving biodiversity [5]. The PHD may also help reduce social inequalities by promoting affordable foods like whole grains, vegetables, and legumes [5], thus improving health and autonomy of vulnerable populations [6]. However, large-scale effectiveness requires supportive policies such as subsidies for healthy foods and fair food systems [7].

Adopting the PHD faces cultural and social resistance, the prevalence of animal-based proteins, and traditional eating habits, complicating the shift to sustainable practices. While studies have explored consumer behaviors and the role of meat substitutes [8], none have specifically addressed populations’ knowledge, attitudes, and behaviors (KAB) regarding the PHD. Transforming food systems is complex, shaped by political, social, and cultural factors [9]. Investigating KAB could therefore be crucial for developing effective, evidence-based interventions for dietary transitions [10]. Such framework enables to analyze how knowledge influences attitudes and drives behaviors [11]. People might know that plant-based diets are healthy, but cultural norms, cost, or taste concerns often hinder change [12–14]. Even with favorable attitudes, limited access to PHD-approved foods or cooking knowledge can prevent action [13].

Applying the KAB methodology within the PHD context may provide nuanced insight into these challenges. It can guide education campaigns, policies and systemic changes to support adoption [4]. Moreover, it aligns with national initiatives such as the French National Environmental Health Plan and the French National Nutrition and Health Program [15], as well as international guidelines from the WHO [16] and, the Food and Agriculture Organization (FAO) [6], which promote sustainable diets for health, social, sociocultural and environmental outcomes.

To understand such dynamics across groups, this study sampled a heterogeneous cross-section of the French population—patients, healthcare professionals (specifically Hospices Civils of Lyon (HCL—Lyon Civil Hospitals employees), and farmers-bakers’ customers—to capture broad perspectives. Healthcare professionals are key agents of health promotion, yet their own dietary habits remain underexplored [17]. Patients, often managing chronic conditions, benefit greatly from improved nutrition [18]. Customers of local producers reflect traditional consumption patterns [19]. This strategic sampling enables a multidimensional analysis of KAB across socioprofessional and ecological settings in France.

This study aims to assess KAB regarding the PHD among these French populations to identify barriers and facilitators of sustainable dietary behaviors. This will inform targeted strategies to promote healthy and sustainable food transitions aligned with public health and environmental goals.

## Methods

### Study design

This study is a part of the BIOQUALIM protocol published by Murat-Ringot et al. (2024) [20]. This study was a cross-sectional survey based on a structured questionnaire administered between the 15th April 2024 and the 15th July 2024, and reported according to the Checklist for Reporting Of Survey Studies (CROSS) [21](Supplementary Table 1). It analyzed KAB regarding PHD in a general population using a specific on-purpose questionnaire.

### Participants

The included population was: (i) people over 18 years old, (ii) able to read and understand the French language, (iii) users (patients and professionals) of the Hospices Civils of Lyon (HCL - Lyon Civil Hospitals) or, (iv) farmers-bakers’ customers.

### Procedure

#### Sample method

The population was recruited based on a convenience sampling methodology. The questionnaire was distributed both in print and online to users of the central kitchen of the HCL and to farmers-bakers’ customers.

A link and QR code were provided for each questionnaire, with a paper version available for patients and farmers-bakers’ customers. For HCL staff, the survey was shared via email from General Management and through a pop-up on staff computers. Flyers were distributed with meal trays and displayed in waiting rooms for patients and healthcare workers, while customers at farms and markets accessed the survey through flyers and posters.

#### Sociodemographic, dietary and geographic variables

Sociodemographic and dietary variables were collected through predefined closed-ended questions included in the questionnaire. Profession was self-reported according to standard French socio-professional categories. Main diet was collected using fixed response options (omnivorous, flexitarian, vegetarian, vegan, pesco-vegetarian). Weekly food budget was self-declared using predefined brackets (1–50 €, 51–100 €, 101–150 €, > 150 €). Household size and age group were also self-reported.

Geographic variables were included as contextual covariates to explore potential spatial disparities in KAB scores. These variables were defined at the municipality level and included population density, average income per inhabitant, and the Geographic Classification for Health studies (GeoClasH), a composite index built from 10 variables describing physical, social, and medical environments [22]. Average income quintiles were not self-reported but derived from INSEE (French National Institute of Statistics and Economic Studies) municipal-level data and assigned to each participant according to their municipality of residence. Income quintiles corresponded to the national distribution of municipal median disposable incomes (Q1 = €15,015–18,072; Q2 = €18,110–19,320; Q3 = €19,480–20,732; Q4 = €20,774–22,974; Q5 = €23,021–36,562). Population density was categorized into national quintiles.

#### Development and validation of the KAB questionnaire on PHD

##### Questionnaire design and validation

A structured questionnaire in French has been developed to assess KAB towards PHD. The methodology followed the recommendations of the WHO [23]. The conceptual framework of the questionnaire is presented in Supplementary Fig. 1.

To develop the questionnaire, two researchers performed a literature review to identify studies assessing PHD-related KAB. Based on this, questions were selected, adapted, or created, inspired in particular by the Food Choice Questionnaire [24]. This first version included 136 closed items. Validation followed Andrade et al. (2020) [25], incorporating facial (surface) and content validity. Facial validation assesses purpose alignment, while content validation ensures completeness, relevance and clarity. An expert committee (1 nutritionist, 1 dietician, 2 plant-based diet specialists, and 1 general practitioner) evaluated each item, suggesting revisions where needed. After calculating the content validity index, the revised version was reviewed again. Cognitive debriefing with 20 people followed, leading to final refinements. The approved version included 9 close-ended thematic question blocks, each composed of multiple scored items, for a total of 88 scored items (Knowledge: 36 items, Attitude: 21 items, Behavior: 31 items)(Supplementary KAB questionnaire).

##### Construction of the KAB score

After validation of the questionnaire, a scoring system was developed to quantify knowledge, attitude, and behavior (KAB) toward the PHD. To ensure item relevance and scoring objectivity, a group of experts (1 public health specialist, nutritionist, 2 dieticians, and 2 general practitioners) completed the questionnaire. The Delphi method [26] was used to reach consensus on correct answers. Experts compared responses and resolved discrepancies through discussion. KAB scores were calculated using + 1 for PHD-aligned answers and − 1 otherwise. The total KAB score (sum of these scores) measured overall knowledge, attitude, and behavior toward the PHD. The knowledge score (K) was based on 36 items (range: -36 to + 36), the attitude score (A) on 11 items (range: -11 to + 11) and the behavior score (B) on 31 items (range: -31 to + 31). All scores were normalized to a 0–40 scale using the following formula:

$$\:\mathrm{Normalized\:Score}=\frac{\mathrm{Number\:of\:correct\:answers}}{\mathrm{Total\:items}}\times\:40$$

The scores were categorized as follows: 0–23 = inadequate, 24–29 = marginal, and 30–40 = adequate. The total KAB score, ranging from 0 to 120, followed this classification: 0–70 = inadequate, 71–88 = marginal, and 89–120 = adequate. These cut-offs were adapted from thresholds commonly used in validated health literacy scoring systems, which classify scores into three levels reflecting insufficient, borderline, and adequate literacy. In particular, our thresholds correspond proportionally to those of the Test of Functional Health Literacy in Adults [27] and Oral Health Literacy Instrument [28, 29], which define inadequate literacy as scores below 60%, marginal literacy as 60–74%, and adequate literacy as 75–100%. To align these categories with our normalized 0–40 scale, proportional thresholds were derived using a rule-of-three transformation.

##### Survey administration

Participants completed the online questionnaire using LimeSurvey (Version 6.5.7) with anonymized responses. To prevent multiple submissions, cookies were enabled, a CAPTCHA (acronym for ‘Completely Automated Public Turing test to tell Computers and Humans Apart’) was required to secure question-and-answer authentication and an individual token access has been set up to enable secure connection. Participants could also pause and resume the questionnaire if needed.

### Outcomes

The primary outcome of the study was to describe the levels of K, A, B, and the combined KAB scores related to PHD among healthcare professionals, patients, and farmers/bakers’ customers in France.

The secondary outcomes were:

- To identify the sociodemographic, professional, and/or clinical determinants associated with inadequate levels of K, A, B, and KAB regarding PHD.
- To compare the distribution of these scores across relevant subgroups.

### Statistical analysis

#### Sample size

Considering a population of more than 8.000 professionals and 2.000 patients, all users of the central kitchen of the HCL, with a confidence level of 95% and a margin of error of 5%, a return of 383 questionnaires was estimated as representative of the population. For the customers of the 5 farmers-bakers, the estimated population consisted of 500 individuals residing in the Rhône and Loire regions, with a confidence level of 95% and a margin of error of 5%, a return of 218 questionnaires was estimated as representative of the population.

#### Analysis

A descriptive analysis summarized the participants’ sociodemographic characteristics. Stratification by selected covariates (e.g., age group) explored subgroup differences.

Pearson correlation coefficients tested relationships between scores, with thresholds ranging from null or low (0.00- 0.25), low (0.26–0.49), moderate (0.50–0.69), high (0.70–0.89) to very high (0.90-1.00) [30]. 

Binary logistic regressions (univariate and multivariate) estimated odds ratios (OR) to assess associations between sociodemographic, dietary factors and the probability of having inadequate scores. Focusing on inadequate scores was prioritized for its public health relevance and clearer logistic modeling, given the small and heterogeneous nature of the adequate group. Multivariate logistic regression models were adjusted for all covariates described above, including age group, gender, population group (HCL employees, patients, farmers-bakers’ customers), profession, household size, main diet, weekly food budget, population density quintiles, income quintiles, and GeoClasH classification.

*P*-values and 95% confidence intervals (CI) supported interpretation. A backward stepwise regression using the Likelihood Ratio (LR) method refined each model, removing non-significant variables and identifying key predictors of inadequate K, A, B, and KAB scores.

### Ethics

This study was approved by the HCL local ethics committees (n° IRB00014232 2024 06 13_12 - June 19, 2024). It was conducted per the Declaration of Helsinki and the reference methodology MR-004 of the French National Commission on Information Technology and Liberties (CNIL). All participants gave their informed consent.

### Role of the funding source

The funder of the study had no role in study design, data collection, data analysis, data interpretation, or writing of the report.

## Results

### Sociodemographic characteristics of participants

**Fig. 1:**
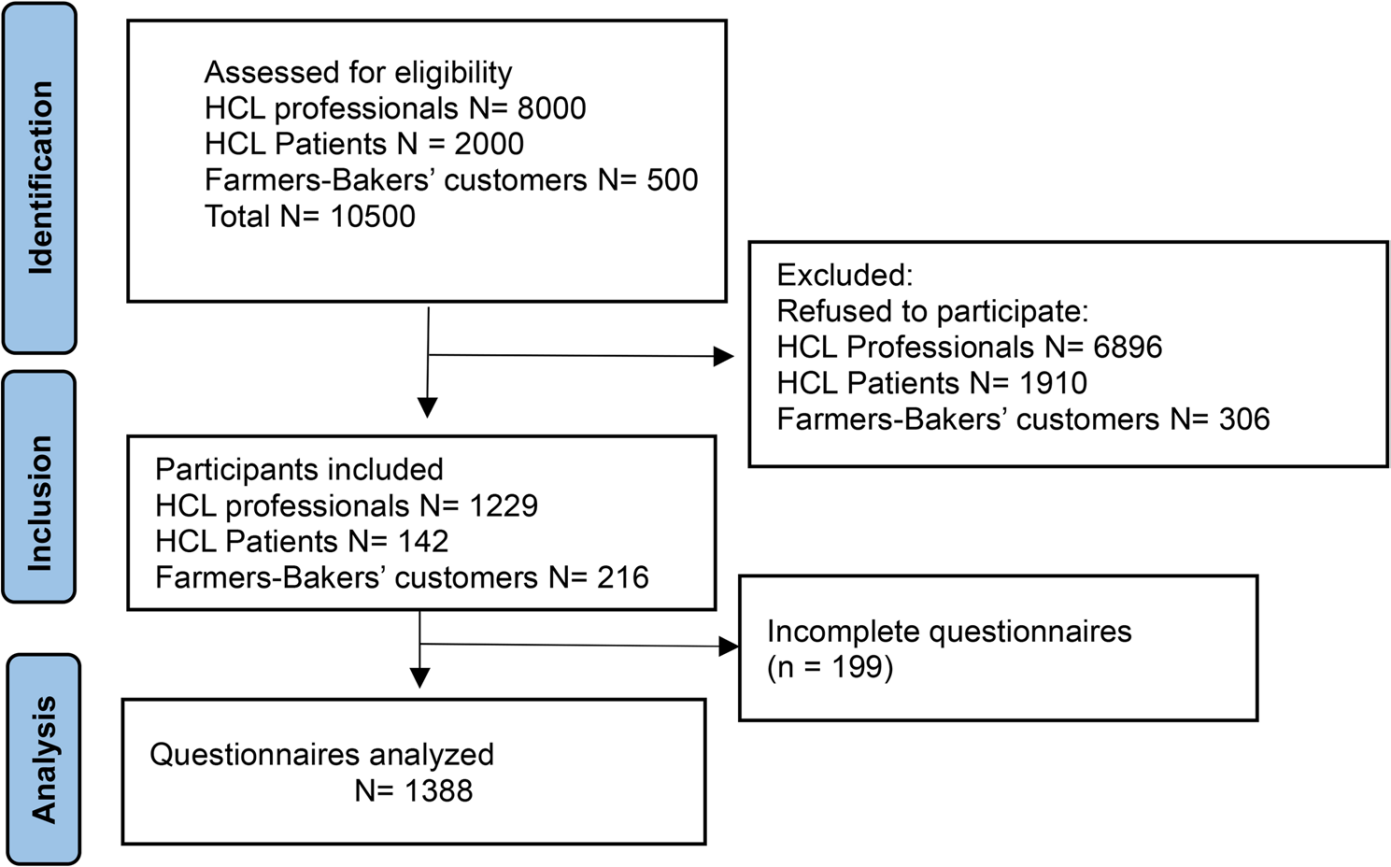
Flowchart of the study

Figure 1 represents the flowchart of the study. 10,500 persons were assessed for eligibility, among whom 9112 refused to participate. Finally, 1388 questionnaires were included.

Supplementary Table 2 presents the sociodemographic, geographic and clinical characteristics of the participants. Among the 1388 participants, 6.4% were HCL patients (*n* = 90), 79.6% were HCL professionals (*n* = 1104) and 14% were farmers-bakers’ customers (*n* = 194). Participants had a mean age of 45.73 (SD 12.8), 79.0% were female (*n* = 1096), and the household consisted of a mean of 2.8 (SD 1.30) people. 56.8% of participants (*n* = 788) had an intermediate profession. The largest proportion (32.9%, *n* = 457) of participants live in the highest density zones ranging from 4071.24 to 11623.75 people per km2. 60.7% of participants (*n* = 842) were omnivores. Household weekly food budget most commonly ranged from €51–100 (31.9%, *n* = 443).

### Knowledge, attitude, behavioral, and KAB scores of all participants

Supplementary Table 3 presents descriptive statistics for K, A, B, and KAB scores. KAB score ranged from 22.54 to 102.71, with a mean of 63.60 (SD 13.45). For the K section, the participants obtained a mean score of 29.60 (SD 6.51), while for the A section, the mean score was 17.03 (SD 7.88) and for the B section the mean score was 16.97 (SD 4.29). An adequate level of KAB was achieved by only 3% of participants (*n* = 41), whereas 68.9% (*n* = 956) had an inadequate level.

### Analysis of the correlation between K, A, B and KAB scores

Supplementary Table 4 presents the matrix of correlations between K, A, B and KAB scores. No correlation was observed between K and A or B. A low correlation was observed between A and B. K and B were moderately correlated to KAB while A was highly correlated to KAB.

### Identification of determinants of PHD inadequate K, A, B, and KAB scores

**Fig. 2:**
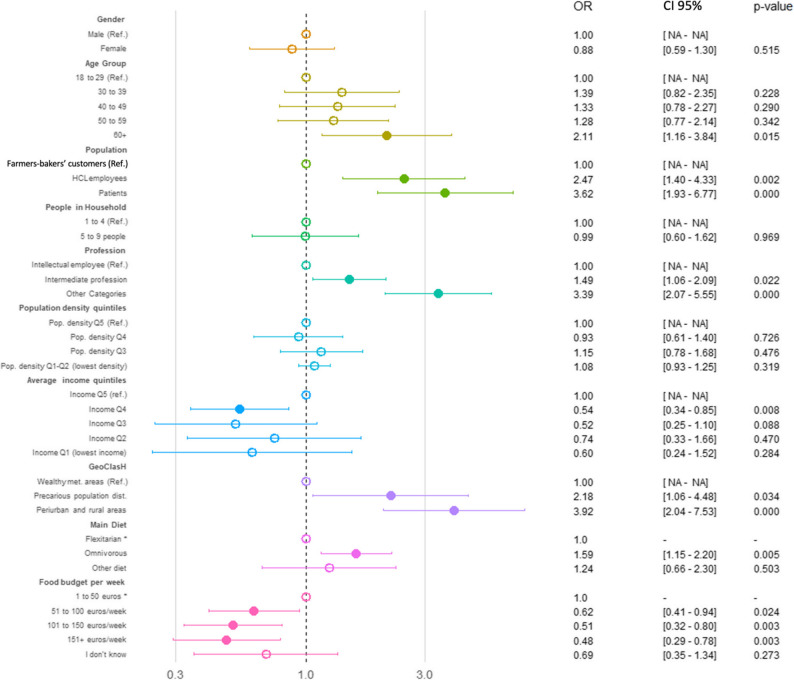
Binary multivariate logistic regression identifying the determinants of inadequate K regarding PHD. An empty circle indicates a *p*-value greater than 0.05, while a filled circle indicates a p-value of 0.05 or less. *CI* Confidence Interval, *OR* Odds Ratio, *Ref* Reference group

**Fig. 3:**
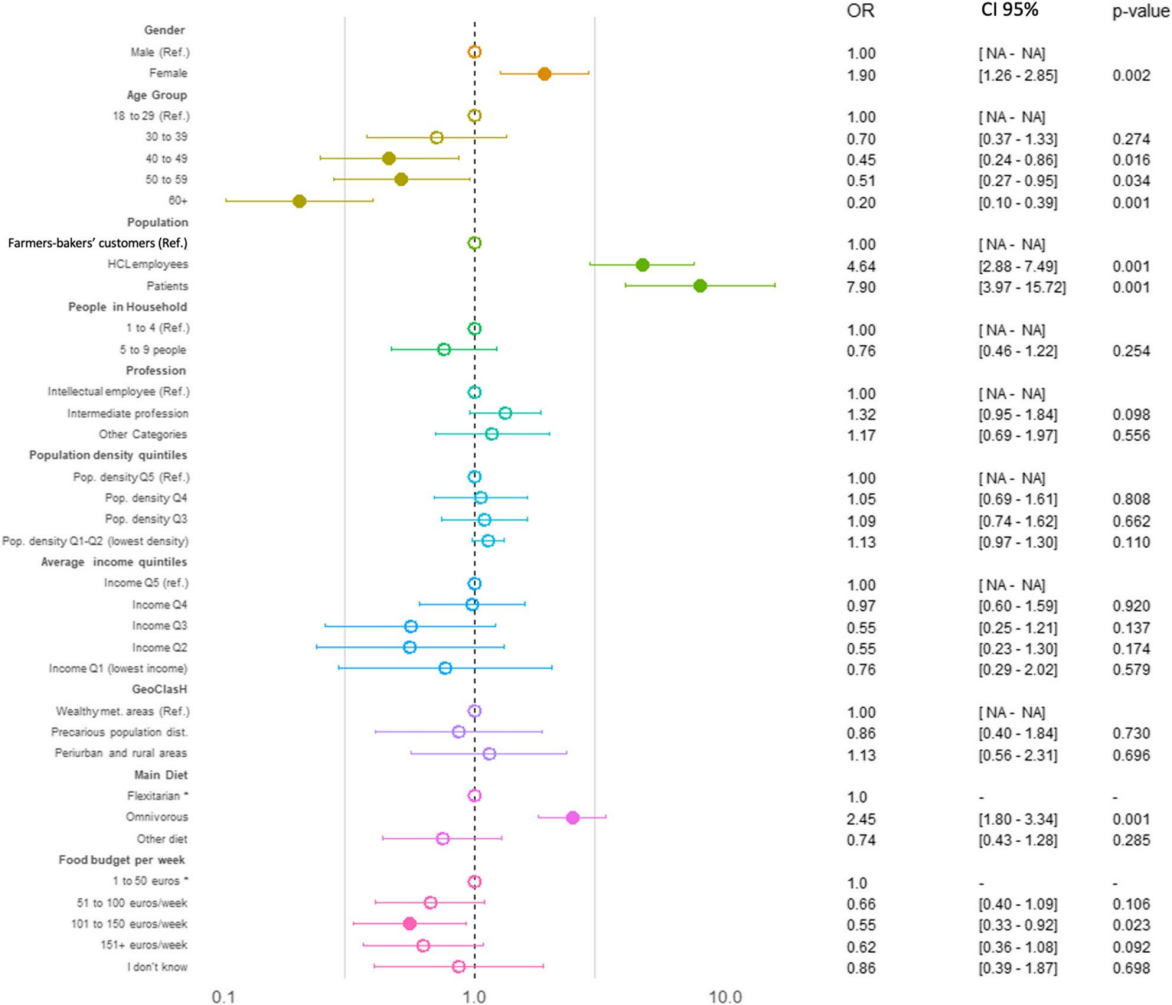
Binary multivariate logistic regression identifying the determinants of inadequate A regarding PHD. An empty circle indicates a p-value greater than 0.05, while a filled circle indicates a *p*-value of 0.05 or less. *CI* Confidence Interval, *OR* Odds Ratio, *Ref* Reference group

**Fig. 4:**
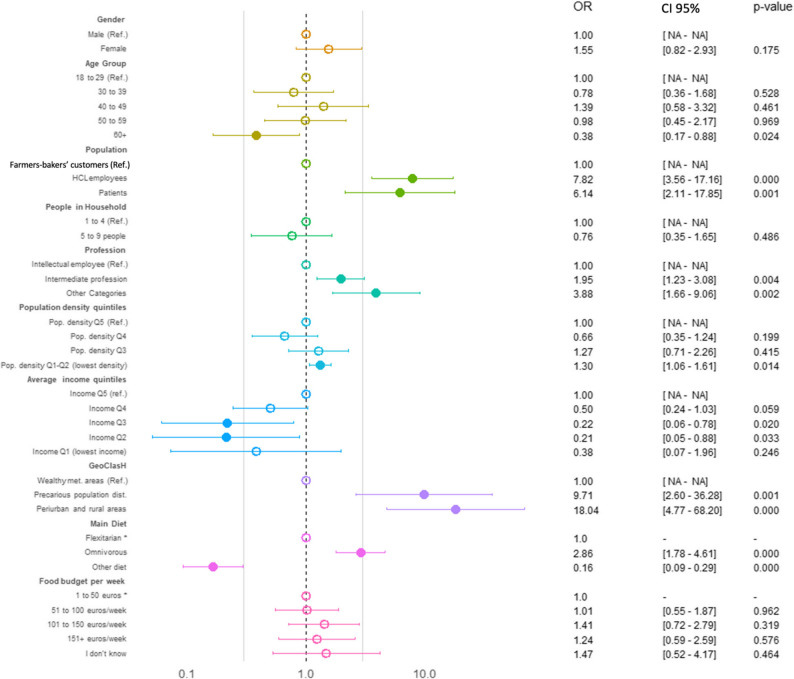
Binary multivariate logistic regression identifying the determinants of inadequate B regarding PHD. An empty circle indicates a p-value greater than 0.05, while a filled circle indicates a p-value of 0.05 or less. *CI* Confidence Interval, *OR* Odds Ratio, *Ref* Reference group

**Fig. 5:**
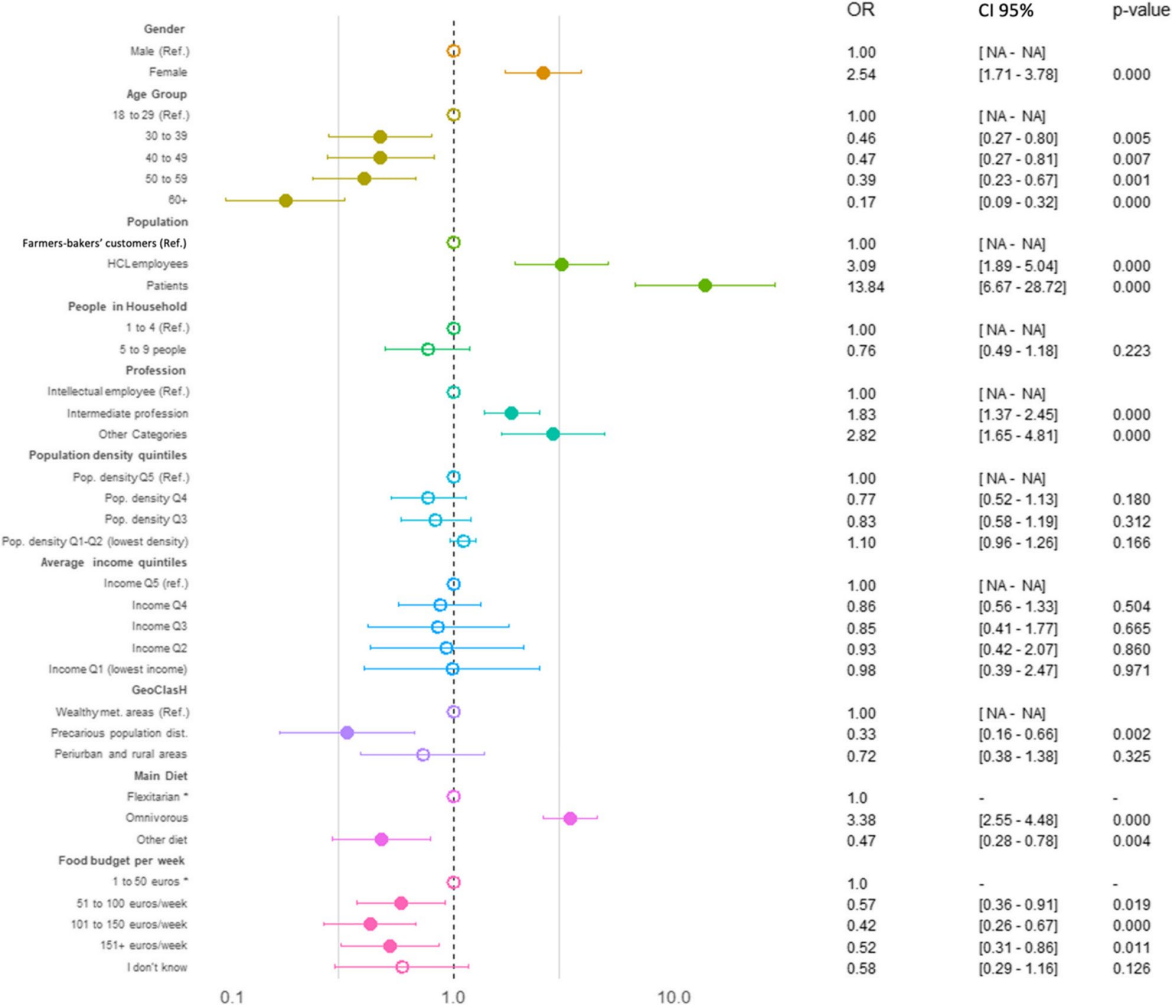
Binary logistic regression identifying the determinants of inadequate KAB regarding PHD. An empty circle indicates a p-value greater than 0.05, while a filled circle indicates a p-value of 0.05 or less. *CI* Confidence Interval, *OR* Odds Ratio, *Ref* Reference group

The results of the univariate logistic regression are presented in Supplementary Table 5. The multivariate logistic regression analysis identified several significant and independent determinants of PHD inadequate K, A, B, and KAB scores (Figs. 2, 3, 4 and 5 and Supplementary Table 6).

For K inadequate score, professional category, population group, geographic classification, diet, and food budget emerged as robust determinants (Fig. 2 and Supplementary Table 6). Specifically, individuals in “Other Categories” professions had higher OR of inadequacy (OR = 3.39, *p* < 0.001), while participants of precarious population districts faced significantly elevated risk (OR = 2.18, *p* = 0.034). The considered group was particularly impactful: compared to farmers-bakers’ customers, both HCL employees (OR = 2.47, *p* = 0.001) and patients (OR = 3.62, *p* < 0.001) were more likely to experience K inadequacy. Dietary habits played a notable role, with omnivorous participants showing increased odds (OR = 1.59, *p* = 0.005). Moreover, a higher weekly food budget, particularly above €150, served as a significant protective factor (OR = 0.48, *p* = 0.003). In contrast, variables such as age, income level, and population density were not predictive values after adjustment.

In the case of A inadequate score, the multivariate model highlighted dietary pattern and population group as key independent determinants (Fig. 3 and Supplementary Table 6). Omnivorous individuals were associated with higher odds of inadequacy (OR = 2.45, *p* = 0.001). Occupational status was determinant: HCL employees (OR = 4.64, *p* = 0.001) and patients (OR = 7.90, *p* = 0.001) demonstrated significantly less adapted attitudes towards PHD compared to farmers-bakers’ customers. Other factors like income and geographic classification, which showed protective trends in univariate analysis (Supplementary Table 4), were no longer significant after adjustment, indicating they may be confounded by stronger covariates.

For B inadequate score, age emerged as a protective determinant, with individuals aged 60 and over experiencing significantly lower risk of B inadequacy (OR = 0.38, *p* = 0.024) (Fig. 4 and Supplementary Table 6). Dietary pattern was a major risk factor, as omnivorous diets were associated with increased inadequacy (OR = 2.86, *p* < 0.001). Interestingly, professional categories that initially appeared protective in univariate analysis showed increased risk after adjustment: “Intermediate Professions” (OR = 1.95, *p* = 0.004) and “Other Categories” (OR = 3.88, *p* = 0.002). Additionally, people living in cities with moderate income levels (Q2–Q3) have a less inadequate behavior towards PHD, in comparison to residents of precarious districts (OR = 9.71, *p* = 0.001) and “Other areas” (OR = 18.04, *p* < 0.001) who experienced elevated risk. Population density quintiles also gained significance, with individuals in low-density areas (Q1–Q2) having higher odds of inadequacy (OR = 1.30, *p* = 0.014).

The KAB inadequate score was associated with the combined effects of dietary habits, sociodemographic status, and environment (Fig. 5 and Supplementary Table 6). Older age (60+) was strongly protective (OR = 0.17, *p* < 0.001), while omnivorous diets remained a potent risk factor (OR = 3.38, *p* = 0.024). Patients had the highest odds of inadequacy (OR = 13.84, *p* < 0.001), followed by HCL employees. Female participants exhibited significantly greater risk (OR = 2.54, *p* < 0.001). Professional categories showed a similar inversion, with “Intermediate Professions” (OR = 1.83, *p* < 0.001) and “Other Categories” (OR = 2.82, *p* < 0.001) indicating higher risk after adjustment. Notably, a food budget exceeding €100/week offered consistent protection (101–150 €/week: OR = 0.42; >150 €/week: OR = 0.52), while income quintiles were no longer significant predictors.

## Discussion

This cross-sectional study is the first to evaluate the levels of KAB regarding the PHD in a French population comprising healthcare professionals, patients, and farmers-bakers’ customers—groups strategically selected for their diverse roles within the food and health systems.

Importantly, the study does not aim to represent the French population as a whole. The sampling strategy intentionally focused on three groups with a particular relationship to nutrition and health: healthcare professionals, patients, and customers of farmers-bakers. Healthcare professionals typically display higher nutritional knowledge and greater exposure to dietary recommendations through their clinical roles [31, 32]. Patients are frequently targeted by nutritional counselling, especially when managing chronic diseases [18]. Customers of farmers-bakers often engage in short food-supply chains, a behavior associated with stronger environmental motivations and more sustainable dietary patterns [19]. For these reasons, our findings should be interpreted as reflecting the KAB patterns of these specific subgroups rather than the general French population.

In this specific context, despite growing recognition of sustainable diets’ health and ecological benefits, only 3% of participants achieved full KAB adequacy, with critical gaps particularly evident between knowledge (61%) versus attitude (3.5%) and behavior (0.2%).

Considering that 79.6% of respondents were healthcare professionals—who generally present higher health literacy—the relatively low Attitude and Behavior scores require specific consideration. Prior research reports a persistent “knowledge–behavior gap” among healthcare professionals, who often struggle to translate their expertise into personal practices. Occupational constraints—including irregular schedules, shift work, high fatigue, and limited time for meal planning—have been shown to adversely affect dietary behaviors in this population [33, 34]. In addition, environmental constraints within healthcare institutions, including the food environment of hospitals and workplaces, may limit access to healthy or sustainable options and thereby hinder behavior change [35, 36] Thus, the low Attitude and Behavior scores observed in our study align with the broader literature and likely reflect contextual constraints rather than a contradiction with their health promotion role.

The discrepancy between knowledge and observed behavior corroborates the persistent “knowledge–action gap” identified in dietary transition research across Europe. Similar to a 2024 review by Bogueva et al., many consumers demonstrate high cognitive awareness of sustainable eating principles but exhibit resistance to translating into practice due to sociocultural or logistical barriers [14]. This aligns with the poor attitude and behavior adequacy scores observed in our cohort, suggesting that cognitive understanding of the PHD alone is not sufficient to influence food choice dynamics. In addition, a recent study by Viroli et al. (2023) further supports this disjunction, showing that although plant-based diets are positively associated with health and environmental values, taste preferences, affordability perceptions, and culinary unfamiliarity often prevent uptake, particularly among omnivorous populations [37].

Sociodemographic and economic determinants such as omnivorous dietary patterns, female gender, and patient status were identified as significant risk factors for KAB inadequacy. Our sample was predominantly female, reflecting the gender distribution of HCL professionals. Although this imbalance may influence effect estimates, the association between female gender and inadequate KAB remained significant after multivariate adjustment, suggesting that this finding is not solely due to sample skewness.

The observation that female gender was associated with inadequate KAB is consistent with findings of Baldemor et al. (2024) [38], who observed greater susceptibility to dietary misinformation and fad diets among general medical populations and younger women in developing countries. Moreover, recent research indicates that gender differences in sustainable diet adherence are common. Women frequently report stronger environmental and health-related motivations but also greater perceived barriers—including cost, cultural expectations, family-related food responsibilities, and sensory constraints—that may limit actual behavioral change [13, 14, 39]. Thus, the observed association may reflect broader sociocultural dynamics rather than a methodological artefact.

In contrast, older adults (≥ 60 years) and participants with higher weekly food budgets (>€100) demonstrated significantly better KAB scores, which may be linked to increased health awareness and greater dietary agency.

Our results report significant lower risk of KAB inadequacy for participants from precarious districts which may reflect targeted nutritional outreach or institutional programs — a hypothesis also hinted at in WHO’s 2021 report on plant-based diet uptake in underserved European communities [16]. However, this result should be treated with caution, as these same areas also show contradictory results, with a significantly higher risk of inadequacy for K and B scores.

In Europe, a similar KAB analysis conducted in the UK and the Netherlands [11] found comparable trends — strong positive attitudes in urban populations failed to materialize in sustained behavior change without concurrent changes in food environments and social norms. These studies collectively highlight the necessity of multilevel interventions, including fiscal policies, educational tools, and community-based support.

The discrepancy between high levels of knowledge and low levels of observed attitudes and behavioral adherence observed underlined the limitations of conventional health promotion strategies. Truman and Elliott highlighted the fact that food literacy is influenced by a complex interaction of individual, social and environmental factors [40]. Informational campaigns alone appear insufficient to drive meaningful dietary change. As highlighted in the EAT-Lancet 2.0 recommendations [4] and the FAO/WHO guidelines [2] on sustainable diets, a successful dietary transition requires comprehensive structural and cultural support. In line with these frameworks, effective strategies should include:

- Economic levers, such as financial incentives and targeted subsidies to improve the affordability of sustainable food options;
- Reform of institutional food environments, particularly within healthcare settings, to model and reinforce sustainable dietary practices;
- Culinary education initiatives, designed to address practical barriers by building food preparation skills aligned with plant-rich diets, also emphasize the use of herbs and spices to enhance flavor and appeal, aiming to attract omnivores toward more plant-based eating habits.
- Culturally sensitive communication strategies, tailored to specific populations—particularly omnivores and women, who in our study exhibited greater vulnerability to behavior-knowledge misalignment and may face unique sociocultural pressures related to dietary change and body image.

### Limitations

First, the use of convenience sampling, particularly targeting healthcare professionals, patients, and farmers-bakers’ customers—populations likely to be more responsive to the principles of the PHD—may limit the generalizability of the findings. Second, the sample is disproportionately composed of women, which may influence the overall responses and limit the representativeness of the data. However, this proportion is in accordance with that of HCL staff (75% women) and patients (57% women). Third, the reliance on self-reported data, especially regarding behavior-related items, introduces the possibility of social desirability bias. Fourth, the cross-sectional design provides only a snapshot in time, preventing any conclusions about causality. Nonetheless, the study offers a rigorous and innovative assessment of PHD-related KAB in France, supported by a validated instrument and a large, heterogeneous sample. Fifth, the classification of scores as “inadequate”, “moderate” and “adequate” was based on previously published classification schemes, such as those for literacy. However, using different cut-off ranges might have led to different results. Sixth, while the KAB model was used to assess the French population’s stance toward the PHD, alternative approaches—such as practice theories—could also have been employed. These theories focus on social practices as organized sets of actions and discourses, shaped by meanings, norms, and emotions directed toward specific goals [41]. 

## Conclusions

This study reveals a significant gap between knowledge and actual behavior regarding the PHD in France. Although participants demonstrated knowledge regarding several PHD-related principles, practical adoption remains very limited, especially among omnivores, and socioeconomically vulnerable groups. These findings underscore the need for strategies that go beyond education—incorporating structural, economic, and cultural interventions—to support a fair and widespread transition to sustainable diets, as advocated by EAT–Lancet 2.0 and FAO/WHO.

## Supplementary Information

Supplementary Material 1.

## Data Availability

Deidentified data are available upon request to the corresponding author.

## Abbreviations

FAO: Food and Agriculture Organization
HCL: Hospices Civils of Lyon
KAB: Knowledge Attitude Behavior
NCD: Noncommunicable Diseases
PHD: Planetary Health Diet
WHO: World Health Organization
