# Supplementary material for: Knowledge, attitudes, and behaviors toward the planetary health diet in France: a survey among patients, healthcare professionals, and farmers-bakers’ customers
Source: Nutr J. 2026 Feb 27;25:35. doi: 10.1186/s12937-026-01300-2 (PMC13045154; doi:10.1186/s12937-026-01300-2)
Supplement: Supplementary file 1 — Supplementary Material 1. [file 12937_2026_1300_MOESM1_ESM.docx]

**Supplementary files**

**Table 1.** Checklist for Reporting of Survey Studies (CROSS)

| **Section/topic** | **Item** | **Item description** | **Reported on page #** |
| --- | --- | --- | --- |
| **Title and abstract** | | | **1** |
| Title and abstract | 1a | State the word “survey” along with a commonly used term in title or abstract to introduce the study’s design. | 1 |
|  | 1b | Provide an informative summary in the abstract. covering background. objectives. methods. findings/results. interpretation/discussion. and conclusions. | 1 |
| **Introduction** | | |  |
| Background | 2 | Provide a background about the rationale of study. what has been previously done. and why this survey is needed. | 2 |
| Purpose/aim | 3 | Identify specific purposes. aims. goals. or objectives of the study. | 2 |
| **Methods** | | | **3** |
| Study design | 4 | Specify the study design in the methods section with a commonly used term (e.g.. cross-sectional or longitudinal). | 3 |
|  | 5a | Describe the questionnaire (e.g.. number of sections. number of questions. number and names of instruments used). | 3 |
| Data collection methods | 5b | Describe all questionnaire instruments that were used in the survey to measure particular concepts. Report target population. reported validity and reliability information. scoring/classification procedure. and reference links (if any). | 3 |
|  | 5c | Provide information on pretesting of the questionnaire. if performed (in the article or in an online supplement). Report the method of pretesting. number of times questionnaire was pre-tested. number and demographics of participants used for pretesting. and the level of similarity of demographics between pre-testing participants and sample population. | 4 |
|  | 5d | Questionnaire if possible. should be fully provided (in the article. or as appendices or as an online supplement). | 19 |
| Sample characteristics | 6a | Describe the study population (i.e. background. locations. eligibility criteria for participant inclusion in survey. exclusion criteria). | 4 |
|  | 6b | Describe the sampling techniques used (e.g.. single stage or multistage sampling. simple random sampling. stratified sampling. cluster sampling. convenience sampling). Specify the locations of sample participants whenever clustered sampling was applied. | 5 |
|  | 6c | Provide information on sample size. along with details of sample size calculation. | 3 |
|  | 6d | Describe how representative the sample is of the study population (or target population if possible). particularly for population-based surveys. | 3 |
| Survey  administration | 7a | Provide information on modes of questionnaire administration. including the type and number of contacts. the location where the survey was conducted (e.g.. outpatient room or by use of online tools. such as SurveyMonkey). | 4 |
|  | 7b | Provide information of survey’s time frame. such as periods of recruitment. exposure. and follow-up days. | 4 |
|  | 7c | Provide information on the entry process:  –>For non-web-based surveys. provide approaches to minimize human error in data entry.  –>For web-based surveys. provide approaches to prevent “multiple participation” of participants. | 5 |
| Study preparation | 8 | Describe any preparation process before conducting the survey (e.g. interviewers’ training process. advertising the survey). | 5 |
| Ethical considerations | 9a | Provide information on ethical approval for the survey if obtained. including informed consent. institutional review board [IRB] approval. Helsinki declaration. and good clinical practice [GCP] declaration (as appropriate). | 5 |
|  | 9b | Provide information about survey anonymity and confidentiality and describe what mechanisms were used to protect unauthorized access. | 5 |
| Statistical  analysis | 10a | Describe statistical methods and analytical approach. Report the statistical software that was used for data analysis. | 6 |
|  | 10b | Report any modification of variables used in the analysis. along with reference (if available). | 7 |
|  | 10c | Report details about how missing data was handled. Include rate of missing items. missing data mechanism (i.e.. missing completely at random [MCAR]. missing at random [MAR] or missing not at random [MNAR]) and methods used to deal with missing data (e.g.. multiple imputation). | 8 |
|  | 10d | State how non-response error was addressed. | 12 |
|  | 10e | For longitudinal surveys. state how loss to follow-up was addressed. | N/A |
|  | 10f | Indicate whether any methods such as weighting of items or propensity scores have been used to adjust for non-representativeness of the sample. | 6 |
|  | 10g | Describe any sensitivity analysis conducted. | 12 |
| **Results** | | | **6** |
| Respondent characteristics | 11a | Report numbers of individuals at each stage of the study. Consider using a flow diagram. if possible. | 6 |
|  | 11b | Provide reasons for non-participation at each stage. if possible. | 8 |
|  | 11c | Report response rate. present the definition of response rate or the formula used to calculate response rate. | 14 |
|  | 11d | Provide information to define how unique visitors are determined. Report number of unique visitors along with relevant proportions (e.g. view proportion. participation proportion. completion proportion). | N/A |
| Descriptive  results | 12 | Provide characteristics of study participants. as well as information on potential confounders and assessed outcomes. | 6 |
| Main findings | 13a | Give unadjusted estimates and. if applicable. confounder-adjusted estimates along with 95% confidence intervals and p-values. | 8 |
|  | 13b | For multivariable analysis. provide information on the model building process. model fit statistics. and model assumptions (as appropriate). | 6 |
|  | 13c | Provide details about any sensitivity analysis performed. If there are considerable amount of missing data. report sensitivity analyses comparing the results of complete cases with that of the imputed dataset (if possible). | 12 |
| **Discussion** | | | **10** |
| Limitations | 14 | Discuss the limitations of the study. considering sources of potential biases and imprecisions. such as non-representativeness of sample. study design. important uncontrolled confounders. | 10 |
| Interpretations | 15 | Give a cautious overall interpretation of results. based on potential biases and imprecisions and suggest areas for future research. | 11 |
| Generalizability | 16 | Discuss the external validity of the results. | 12 |
| **Other sections** | | | **12** |
| Role of funding source | 17 | State whether any funding organization has had any roles in the survey’s design. implementation. and analysis. |  |
| Conflict of interest | 18 | Declare any potential conflict of interest. |  |
| Acknowledgements | 19 | Provide names of organizations/persons that are acknowledged along with their contribution to the research. |  |

**KAB Questionnaire**

**Knowledge**

1. **Which of the following do you consider to be plant-based foods (multiple choices possible)?**

☐ Nuts (peanuts, almonds, pistachios, walnuts, hazelnuts...)

☐ Green vegetables

☐ Lentils

☐ Eggs

☐ Cereals grains

☐ Fish

☐ Dried beans

☐ White meat

☐ Seafood

☐ Oils

☐ Dried peas (split peas, chickpeas...)

☐ Spelt

☐ Tofu

☐ Seeds (sunflower, pumpkin seeds, sesame...)

☐ Rice

☐ Quinoa

☐ Potatoes

☐ Buckwheat

1. **Which of these foods are rich in plant protein (several choices possible)?**

☐ Nuts (peanuts, almonds, pistachios, walnuts, hazelnuts...)

☐ Green vegetables

☐ Lentils

☐ Eggs

☐ Cereals grains

☐ Fish

☐ Dried beans

☐ White meat

☐ Seafood

☐ Oils

☐ Dried peas (split peas, chickpeas...)

☐ Spelt

☐ Tofu

☐ Seeds (sunflower, pumpkin seeds, sesame...)

☐ Rice

☐ Quinoa

☐ Potatoes

☐ Buckwheat

**Attitude**

1. **Where do you mainly do your food shopping? (Maximum of 3 choices)**

☐ Supermarkets and hypermarkets

☐ AMAP (Association for the Preservation of Small-Scale Farming)

☐ Local farms and producers (organic or not)

☐ Specialty stores (organic or not)

☐ Markets (organic or not)

1. **What are your main criteria for choosing food products? (Maximum of 3 choices)**

☐ Perceived effect on your health

☐ Origin

☐ Taste quality

☐ Price

☐ Habit

☐ Product composition (additives, sugar, salt, etc.)

☐ Environmental impact

☐ Product brand

☐ Certified product (organic, Label Rouge, etc.)

☐ Producer remuneration and local employment

☐ Promotions/advertising

☐ Ready meals

☐ Animal welfare

1. **Do you ever buy products from farms near your home?**

**☐** Yes **☐** No

1. **Do you buy organic products?**

☐ Yes, most of the time

☐ Yes, sometimes

☐ No, they're too expensive

☐ No, I can't find them where I shop

☐ No, I am not interested

☐ No, I avoid them

☐ I never buy them

1. **Do you have a vegetable garden at home?**

☐ Yes, it is self-sufficient

☐ Yes, but I also shop for additional items

☐ No, but friends and family regularly supply me with produce from their gardens

☐ No

**Behaviors**

1. **In general, what types of foods do you eat?**

| **FOODS** | **NEVER** | **RARELY** | **OCCASIONALLY** | **FREQUENTLY** | **VERY OFTEN** |
| --- | --- | --- | --- | --- | --- |
| Fresh fruit (apples, bananas, oranges, etc.) |  |  |  |  |  |
| Dried fruit (raisins, dried apricots, prunes, etc.) |  |  |  |  |  |
| Processed fruit (compotes, fruit in syrup, etc.) |  |  |  |  |  |
| Vegetables (zucchini, carrots, tomatoes, green beans, sweet corn, peas, etc.) fresh, frozen, and/or canned |  |  |  |  |  |
| Dried vegetables (lentils, chickpeas, split peas, broad beans, dried beans, lupins, soybeans (tofu, tempeh, textured soy protein, soy steak)) |  |  |  |  |  |
| Refined grains (white bread and crackers, white pasta, white rice, white semolina, etc.) |  |  |  |  |  |
| Semi-whole or whole grain wheat and rice products (whole grain bread and crackers, semi-whole grain pasta, semi-whole grain or whole grain pasta, semi-whole grain or whole grain rice, etc.) |  |  |  |  |  |
| Cereals other than wheat and pseudocereals (spelt, oats, quinoa, millet, barley, buckwheat, rye, sorghum, etc.) |  |  |  |  |  |
| Nuts and seeds (walnuts, hazelnuts, almonds, pistachios, seeds, etc.) |  |  |  |  |  |
| Red meat (beef, pork, veal, mutton, lamb, goat, horse, wild boar, venison, etc.) |  |  |  |  |  |
| White meat (chicken, turkey, rabbit, etc.) |  |  |  |  |  |
| Cold cuts and processed meat (cooked or raw ham, sausages and merguez, blood sausage, andouillette, salami, bacon, corned beef, beef jerky, pâté, rillettes, kebab, meatballs, etc.) |  |  |  |  |  |
| Oily fish (salmon, tuna, mackerel, sardines, herring) |  |  |  |  |  |
| Other fish (cod, sea bass, sea bream, etc.) |  |  |  |  |  |
| Seafood/shellfish (mussels, shrimp, oysters, etc.) |  |  |  |  |  |
| Dairy products (milk, yogurt, cheese, cottage cheese, dairy products in prepared meals) |  |  |  |  |  |
| Eggs (eaten as is or in preparations such as cakes, etc.) |  |  |  |  |  |
| Animal fats (butter, crème fraîche, mayonnaise) |  |  |  |  |  |
| Plant-basedfats (vegetable mayonnaise, vegetable margarine) |  |  |  |  |  |
| Oils rich in ALA (omega-3) (rapeseed oil, walnut oil, flaxseed oil, hemp oil, pumpkin seed oil, etc.) |  |  |  |  |  |
| Oils low in ALA (omega-3) (sunflower oil, peanut oil, olive oil, sesame oil, grapeseed oil) |  |  |  |  |  |
| Starch-based products, processed salty/fatty foods (french fries, chips, snack crackers) |  |  |  |  |  |
| Sugary products (cakes, pastries, cookies, chocolates, candies, sweet breakfast cereals, sweet milk desserts, ice cream, etc.) |  |  |  |  |  |
| Industrially prepared meals (pizzas, pasta, chili, lasagna and other ready-to-heat meals, packaged sandwiches, etc.) |  |  |  |  |  |
| **BEVERAGES** | **NEVER** | **RARELY** | **OCCASIONALLY** | **FREQUENTLY** | **VERY OFTEN** |
| Alcoholic beverages (wine, beer, cider) |  |  |  |  |  |
| Spirits (whiskey, gin, vodka, digestifs, etc.) |  |  |  |  |  |
| Soft drinks (carbonated beverages) |  |  |  |  |  |
| Fruit juices (purchased) |  |  |  |  |  |
| Homemade fruit juices |  |  |  |  |  |
| Non-alcoholic fermented beverages |  |  |  |  |  |

**9. Do you usually eat dried vegetables and/or legumes?**

☐ Never or almost never

☐ Rarely

☐ Sometimes

☐ Often

☐ Always or almost always


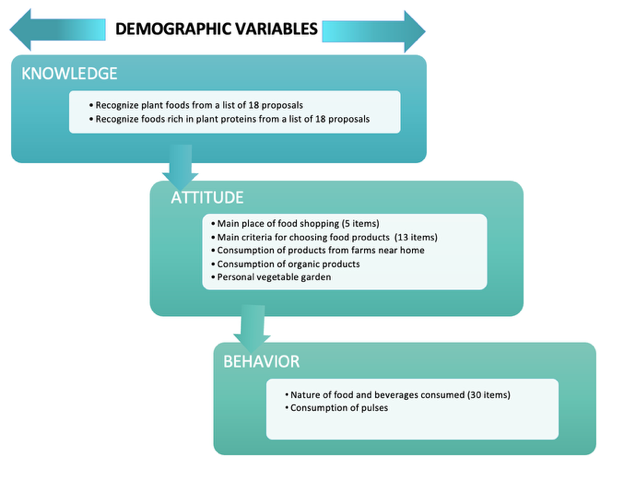


**Figure 1.** Conceptual framework of the questionnaire

**Table 2.** Sociodemographic, geographic and clinical characteristics of the participants.

Due to low numbers, Q1 and Q2 of the density variable were grouped together.

| **Variables All**  **1388** | |
| --- | --- |
| **Gender** | |
| Male | 285 (20.5%) |
| Female | 1096 (79.0%) |
| Non-binary/other | 7 (0.5%) |
| **Age** | |
| 18-29 | 164 (11.8%) |
| 30-39 | 468 (21.9%) |
| 40-49 | 352 (25.4%) |
| 50-59 | 387 (27.9%) |
| 60+ | 181 (13.0%) |
| **Population** | |
| HCL employees | 1104 (79.6%) |
| Patients | 90 (6.4%) |
| Farmers-bakers | 194 (14.0%) |
| **Number of people in the household** | |
| Mean and SD | 2.8 (1.3) |
| **Profession** | |
| Intermediate profession | 788 (56.8%) |
| Senior executive/Intellectual employee | 436 (31.4%) |
| Other Categories | - |
| Farmer | 8 (0.6%) |
| Craftsman/Artisan | 24 (1.7%) |
| Employee | 35 (2.5%) |
| Student | 18 (1.3%) |
| Laborer/Worker | 18 (1.3%) |
| Other | 61 (4.4%) |
| **Population density in quintiles of people per km^2^** | |
| Q1 + Q2 | 283 (20.4%) |
| Q1 (07.71 to 90.42) | 69 (5.0%) |
| Q2 (91.23 to 303.10) | 214 (15,4%) |
| Q3 (306.07 to 1109.28) | 338 (24.4%) |
| Q4 (1119.29 to 3864.58) | 310 (22.3%) |
| Q5 (4071.24 to 11623.75) | 457 (32.9%) |
| **Income per Consumption Unit (CU) in quintiles** (euros per year) | |
| Q1 (15 015 euros to 18 072 euros) | 64 (04.6%) |
| Q2 (18 110 euros to 19 320 euros) | 156 (11.2%) |
| Q3 (19 480 euros to 20 732 euros) | 198 (14.3%) |
| Q4 (20 774 euros to 22 974 euros) | 494 (35.6%) |
| Q5 (23 021 euros to 36 562 euros) | 476 (34.3%) |
| **GeogClasH** | |
| Precarious population districts - n (%) | 441 (31.8%) |
| Wealthy metropolitan areas - n (%) | 797 (57.4%) |
| Other areas | 150 (10.8%) |
| Residential outskirts | 125 (9.0%) |
| Rural margins | 16 (1.2%) |
| Agricultural and industrial plains | 9.0 (0.6%) |
| **Main diet** | |
| Flexitarian | 451 (32.5%) |
| Omnivorous | 842 (60.7%) |
| Other diets | 95 (6.8%) |
| Pesco vegetarian | 23 (1.7%) |
| Vegan | 7.0 (0.5%) |
| Vegetarian | 37 (2.7%) |
| Other | 28 (2.0%) |
| **Household weekly food budget** | |
| 1 – 50 euros/week | 193 (13.9%) |
| 51 – 100 euros/week | 443 (31.9%) |
| 101 – 150 euros/week | 361 (26.0%) |
| > 150 euros week | 319 (22.9%) |
| I don’t know | 72 (5.2%) |

**Table 3.** Descriptive analysis of the scores for the K section, A section, B section, and KAB

|  | K section^a^ | A section^b^ | B section^c^ | KAB^d^ |
| --- | --- | --- | --- | --- |
| Score |  |  |  |  |
| Mean (SD) | 29.60 (6.51) | 17.03 (7.88) | 16.97 (4.29) | 63.60 (13.45) |
| 95% CI | 29.25 to 29.93 | 16.62 to 17.45 | 16.74 to 17.19 | 62.89 to 64.31 |
| Min-Max | 8.89-40 | 0- 36.36 | 5.16- 32.26 | 22.54-102.71 |
| Score level n (%) |  |  |  |  |
| Inadequate^e^ | 312 (22.5%) | 1085 (78.2%) | 1305 (94%) | 956 (68.9%) |
| Marginal^f^ | 229 (16.5%) | 254 (18.3%) | 80 (5.8%) | 391 (28.2%) |
| Adequate^g^ | 847 (61%) | 49 (3.5%) | 3 (0.2%) | 41 (3%) |

^a^Score ranging between 0 and 40; ^b^Score ranging between 0 and 40; ^c^Score ranging between 0 and 40; ^d^Score ranging between 0 and 120; ^e^Between 0 and 23 for K, A, B scores and between 0 and 70 for KAB score; ^f^Between 24 and 29 for K, A, B scores and between 71 and 88 for KAB score; ^g^Between 30 and 40 for K, A, B scores and between 89 and 120 for KAB score.

**Table 4.** Matrix of correlations between score of Knowledge (K), Attitude (A), Behavior (B), and the KAB.

|  | **Knowledge** | **Attitude** | **Behavior** | **KAB** |
| --- | --- | --- | --- | --- |
| **Knowledge** |  |  |  |  |
| r | 1 | 0.254 | 0.111 | 0.669 |
| P value | _a | <.001 | <.001 | <.001 |
| **Attitude** |  |  |  |  |
| r | 0.254 | 1 | 0.380 | 0.830 |
| P value | <.001 | _a | <.001 | <.001 |
| **Behavior** |  |  |  |  |
| r | 0.111 | 0.380 | 1 | 0.560 |
| P value | <.001 | <.001 | _a | <.001 |
| **KAB** |  |  |  |  |
| r | 0.669 | 0.830 | 0.560 | 1 |
| P value | <.001 | <.001 | <.001 | _a |

^a^Not applicable.

**Table 5.** K, A, B and KAB Inadequate scores – Univariate binary logistic regression

| Variables | K score | | | A score | | | B score | | | KAB score | | |
| --- | --- | --- | --- | --- | --- | --- | --- | --- | --- | --- | --- | --- |
|  | OR | IC 95% | p-value | OR | IC 95% | p-value | OR | IC 95% | p-value | OR | IC 95% | p-value |
| Gender |  |  |  |  |  |  |  |  |  |  |  |  |
| Male (Ref.) | 1 |  |  | 1 |  |  | 1 |  |  | 1 |  |  |
| Female | 1.113 | 0.810; 1.530 | 0.487 | 0.901 | 0.660; 1.229 | 0.510 | 1.334 | 0.827; 2.150 | 0.237 | 0.964 | 0.732; 1.269 | 0.793 |
| Age Group |  |  |  |  |  |  |  |  |  |  |  |  |
| 18-29 (Ref.) | 1 |  |  | 1 |  |  | 1 |  |  | 1 |  |  |
| 30-39 | 0.990 | 0.609; 1.609 | 0.968 | 0.635 | 0.361; 1.116 | 0.114 | 0.774 | 0.409; 1.463 | 0.430 | 0.418 | 0.264; 0.663 | <0.001 |
| 40-49 | 1.009 | 0.629; 1.628 | 0.972 | 0.476 | 0.277; 0.818 | 0.007 | 1.984 | 0.965; 4.078 | 0.062 | 0.520 | 0.329; 0.820 | 0.005 |
| 50-59 | 1.014 | 0.637; 1.616 | 0.952 | 0.503 | 0.294; 0.861 | 0.012 | 1.156 | 0.606; 2.204 | 0.660 | 0.432 | 0.276; 0.677 | <0.001 |
| 60+ | 1.729 | 1.042; 2.870 | 0.034 | 0.181 | 0.103; 0.318 | <0.001 | 0.405 | 0.213; 0.772 | 0.001 | 0.221 | 0.135; 0.362 | <0.001 |
| Population |  |  |  |  |  |  |  |  |  |  |  |  |
| Farmers-bakers | 1 |  |  | 1 |  |  | 1 |  |  | 1 |  |  |
| HCL employees | 1.434 | 0.935; 2.198 | 0.098 | 8.233 | 5.917; 11.457 | <0.001 | 4.208 | 2.828; 6.263 | <0.001 | 6.415 | 4.573; 9.001 | <0.001 |
| Patients | 4.332 | 2.428; 7.731 | <0.001 | 8.249 | 4.356; 5.620 | <0.001 | 4.603 | 1.890; 11.210 | 0.001 | 13.719 | 7.163; 26.279 | <0.001 |
| Number of people in the household |  |  |  |  |  |  |  |  |  |  |  |  |
| 1 to 4 (Ref.) | 1 |  |  | 1 |  |  | 1 |  |  | 1 |  |  |
| 5 to 9 | 0.880 | 0.729; 1.062 | 0.182 | 0.866 | 0.725; 1.034 | 0.111 | 1.446 | 1.104; 1.895 | 0.007 | 0.908 | 0.776; 1.062 | 0.227 |
| Profession |  |  |  |  |  |  |  |  |  |  |  |  |
| Intellectual employee (Ref) | 1 |  |  | 1 |  |  | 1 |  |  | 1 |  |  |
| Intermediate profession | 0.617 | 0.504; 0.754 | 0.001 | 0.577 | 0.480; 0.693 | 0.001 | 0.538 | 0.418; 0.694 | <0.001 | 0.458 | 0.391; 0.537 | <0.001 |
| Other Categories | 2.171 | 1.835; 2.570 | 0.001 | 0.433 | 0.363; 0.517 | 0.001 | 0.656 | 0.507; 0.850 | 0.001 | 0.695 | 0.592; 0.818 | <0.001 |
| Population density quintiles |  |  |  |  |  |  |  |  |  |  |  |  |
| Pop. density Q5 (Ref.) | 1 |  |  | 1 |  |  | 1 |  |  | 1 |  |  |
| Pop. density Q4 | 1.087 | 0.772; 1.531 | 0.634 | 0.628 | 0.457; 0.861 | 0.004 | 0.739 | 0.480; 1.136 | 0.168 | 0.549 | 0.415; 0.726 | <0.001 |
| Pop. density Q3 | 1.064 | 0.775; 1.461 | 0.700 | 0.948 | 0.690; 1.303 | 0.741 | 1.017 | 0.644; 1.604 | 0.943 | 0.846 | 0.641; 1.116 | 0.237 |
| Pop. density Q1-Q2 (lowest density) | 0.871 | 0.795; 0.954 | 0.003 | 1.100 | 1.004; 1.205 | 0.042 | 0.989 | 0.869; 1.126 | 0.870 | 1.058 | 0.976; 1.146 | 0.172 |
| Income Quintiles |  |  |  |  |  |  |  |  |  |  |  |  |
| Income Q5 (ref.) | 1 |  |  | 1 |  |  | 1 |  |  | 1 |  |  |
| Income Q4 | 0.740 | 0.537; 1.022 | 1,022 | 1.031 | 0.740; 1.437 | 0.856 | 1.017 | 0.663; 1.560 | 0.937 | 0.952 | 0.721; 1.258 | 0.731 |
| Income Q3 | 0.928 | 0.616; 1.399 | 1,399 | 0.375 | 0.258; 0.545 | <0.001 | 0.776 | 0.459; 1.310 | 0.342 | 0.416 | 0.295; 0.587 | <0.001 |
| Income Q2 | 1.307 | 0.860; 1.986 | 1,986 | 0.512 | 0.338; 0.778 | 0.002 | 1.177 | 0.618; 2.241 | 0.620 | 0.534 | 0.367; 0.778 | 0.001 |
| Income Q1 (lowest income) | 1.122 | 0.605; 2.083 | 2,083 | 0.643 | 0.348; 1.187 | 0.158 | 1.605 | 0.558; 4.617 | 0.380 | 0.611 | 0.356; 1.049 | 0.074 |
| GeoClasH |  |  |  |  |  |  |  |  |  |  |  |  |
| Wealthy met. areas (Ref.) | 1 |  |  | 1 |  |  | 1 |  |  | 1 |  |  |
| Precarious population dist. | 1.334 | 0.996; 1.788 | 0.053 | 0.380 | 0.288; 0.500 | <0.001 | 1.037 | 0.707; 1.520 | 0.853 | 0.389 | 0.305; 0.498 | <0.001 |
| Periurban and rural areas | 2.430 | 1.651; 3.576 | <0.001 | 0.659 | 0.429; 1.015 | 0.058 | 3.417 | 1.362; 8.571 | 0.009 | 0.723 | 0.496; 1.053 | 0.091 |
| Main diet |  |  |  |  |  |  |  |  |  |  |  |  |
| Flexitarian (Ref.) | 1 |  |  | 1 |  |  | 1 |  |  | 1 |  |  |
| Omnivorous | 1.555 | 1.153; 2.097 | 0.004 | 2.884 | 2.193; 3.793 | <0.001 | 4.034 | 2.589; 6.286 | <0.001 | 3.767 | 2.942; 4.824 | <0.001 |
| Other diets | 1.147 | 0.641; 2.053 | 0.644 | 1.019 | 0.636; 1.632 | 0.939 | 1 |  |  | 0.750 | 0.480; 1.173 | 0.208 |
| **Food Week Shopping Budget** |  |  |  |  |  |  |  |  |  |  |  |  |
| 1 – 50 €/week (ref.) | 1 |  |  | 1 |  |  |  |  |  | 1 |  |  |
| 51 – 100 €/week | 0.555 | 0.377; 0.816 | 0.003 | 1.025 | 0.675; 1.558 | 0.906 | 1 |  |  | 0.794 | 0.547; 1.154 | 0.227 |
| 101 – 150 €/week | 0.499 | 0.332; 0.749 | 0.001 | 0.876 | 0.572; 1.340 | 0.541 | 0.774 | 0.409; 1.463 | 0.430 | 0.657 | 0.448; 0.962 | 0.031 |
| > 150 €/week | 0.432 | 0.282; 0.663 | <0.001 | 0.851 | 0.551; 1.312 | 0.464 | 1.984 | 0.965; 4.078 | 0.062 | 0.690 | 0.467; 1.020 | 0.063 |
| I don’t know | 0.814 | 0.444; 1.494 | 0.507 | 0.993 | 0.510; 1.935 | 0.985 | 1.156 | 0.606; 2.204 | 0.660 | 0.757 | 0.423; 1.356 | 0.350 |

**Table 6.** Heatmap for K, A, B and KAB scores, from the red (worst score regarding Planetary Health diet) to green (best score regarding Planetary Health diet). * p<0.05

|  | Variables | K - OR | A - OR | B - OR | KAB OR |  |
| --- | --- | --- | --- | --- | --- | --- |
| Gender | Female | **0.88** | **1.90** | **1.55** | **2.54** |  |
| Age group | 30 to 39 | **1.39** | **0.70** | **0.78** | **0.46** |  |
|  | 40 to 49 | **1.33** | **0.45** | **1.39** | **0.47** |  |
|  | 50 to 59 | **1.28** | **0.51** | **0.98** | **0.39** |  |
|  | 60+ | **2.11** | **0.20*** | **0.38*** | **0.17*** |  |
| Population | HCL employees | **2.47*** | **4.64*** | **7.82*** | **3.09** |  |
|  | Patients | **3.62*** | **7.90*** | **6.14*** | **13.84*** |  |
| Household | 5 to 9 people | **0.99** | **0.76** | **0.76** | **0.76** |  |
| Profession | Intermediate profession | **1.49** | **1.32** | **1.95** | **1.83** |  |
|  | Other Categories | **3.39*** | **1.17** | **3.88** | **2.82** |  |
| Density quintiles | Q1+Q2 | **1.08** | **1.13** | **1.30** | **1.10** |  |
|  | Q3 | **1.15** | **1.09** | **1.27** | **0.83** |  |
|  | Q4 | **0.93** | **1.05** | **0.66** | **0.77** |  |
| Income quintiles | Q1 | **0.60*** | **0.76** | **0.38*** | **0.98** |  |
|  | Q2 | **0.74** | **0.55** | **0.21*** | **0.93** |  |
|  | Q3 | **0.52*** | **0.55** | **0.22*** | **0.85** |  |
|  | Q4 | **0.54*** | **0.98** | **0.50** | **0.86** |  |
| GeoClasH | Periurban areas | **3.92*** | **1.13** | **18.04*** | **0.33*** |  |
|  | Precarious population dist. | **2.18*** | **0.86** | **9.71*** | **0.72** |  |
| Main diet | Omnivorous | **1.59** | **2.45** | **2.86** | **3.38** |  |
|  | Other diet | **1.24** | **0.74** | **0.17*** | **0.47*** |  |
| Week Food budget | 51 to 100 euros/week | **0.62** | **0.66** | **1.02** | **0.57** |  |
|  | 101 to 150 euros/week | **0.51*** | **0.55** | **1.41** | **0.42*** |  |
|  | 151+ euros/week | **0.48*** | **0.62** | **1.24** | **0.52*** |  |
|  | I don't know | **0.69** | **0.86** | **1.47** | **0.58** |  |

*Asterisks highlight the most significant results, co
